# Supplementary material for: A Chemerin Peptide Analog Stimulates Tumor Growth in Two Xenograft Mouse Models of Human Colorectal Carcinoma
Source: Cancers (Basel). 2021 Dec 28;14(1):125. doi: 10.3390/cancers14010125 (PMC8750290; doi:10.3390/cancers14010125)
Supplement: Supplementary file 1 [file cancers-14-00125-s001.zip › cancers-1493305-supplementary.pdf]

Supplementary Material

Receptor activation assay

Method

The assay for cellular activation of CMKLR1, GPR1 and CCRL2 by chemerin protein or CG34 was carried out as described before (Kroeze, W., Sassano, M., Huang, XP. et al. PRESTO-Tango as an open-source resource for interrogation of the druggable human GPCRome. Nat Struct Mol Biol 22, 362–369 (2015). https://doi.org/10.1038/nsmb.3014). Briefly, HTLA cells (a HEK293 cell line stably expressing a tTA-dependent luciferase reporter and a β-arrestin2-TEV fusion gene) were maintained in RPMI, 10% FBS, 100 U/ml penicillin, 100 µg/ml streptomycin, 2 µg/ml puromycin and 100 µg/ml hygromycin B in a humidified atmosphere at 37 °C in 5% CO2. For transfection, cells were plated in 96 well plates (day 1). The following day, cells were transfected with one of the cDNAs for human CMKLR1, GPR1 or CCRL2 using JetPEI (PolyPLUS). On day 4, 3.5x drug stimulation dilutions of human recombinant chemerin (R&D Systems) and CG34 (peptides&elephants) were prepared in filter-sterilized assay buffer, which consisted of 20 mM HEPES and 1x HBSS, pH 7.4, and 20 µl was added to each well. On day 5, medium and drug solutions were removed from the wells, and 20 µl/well of Bright-Glo (Promega) diluted 20-fold in assay buffer was added to each well. After incubation for 15–20 min at room temperature, luminescence was counted in a Visen (PerkinElmer) luminescence reader.

Results

In order to demonstrate the pharmacological equivalence of the full-length chemerin protein and the chemerin peptide analog CG34, a cell-based activity signaling assay was performed. The three chemerin receptors CMKLR1, GPR1 and CCRL2 were transfected into HTLA cells and assayed for activation by either chemerin or CG34. Both CMKLR1 and CCRL2 were concentration-dependently activated by both substances in similar ways: the EC_{50} value of chemerin on CMKLR1 was found to be 1.3 nM, and the EC_{50} of CG34 was 1.5 nM, with a slightly higher maximum activity for CG34. Similarly, on GPR1 cells, chemerin had an EC_{50} of 5.7 nM for chemerin and CG34 had an EC_{50} of 11.2 nM, with a slightly higher apparent maximum activity for chemerin. As expected, neither ligand was able to activate CCRL2-transfected cells (Supplementary Figure 1). Taken together, the results of the cell-based signaling assay demonstrated the pharmacological equivalence of full-length chemerin and CG34 on the three chemerin receptors CMKLR1, GPR1 and CCRL2.

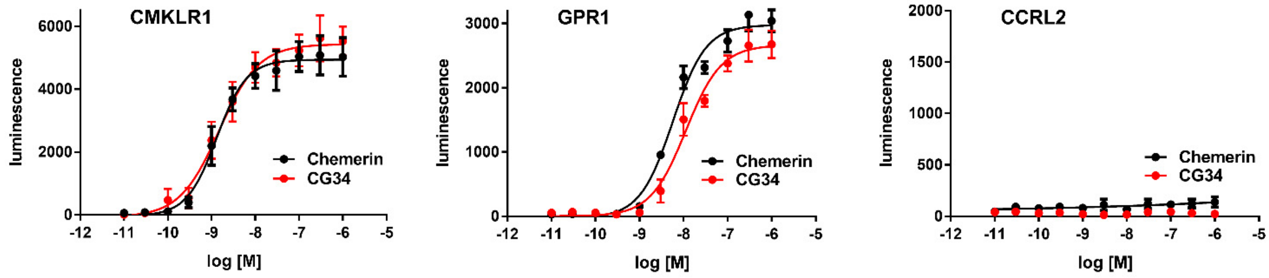

Figure S1. Pharmacological activity of the human recombinant full-length chemerin protein and of the peptide analog CG34 in a cell-based PRESTO-Tango assay in HTLA cells transfected with the human cDNAs of either CMKLR1, GPR1 or CCRL2 (*n*=3 independent experiments performed in triplicate, mean ± S.E.M.).

Treatment study

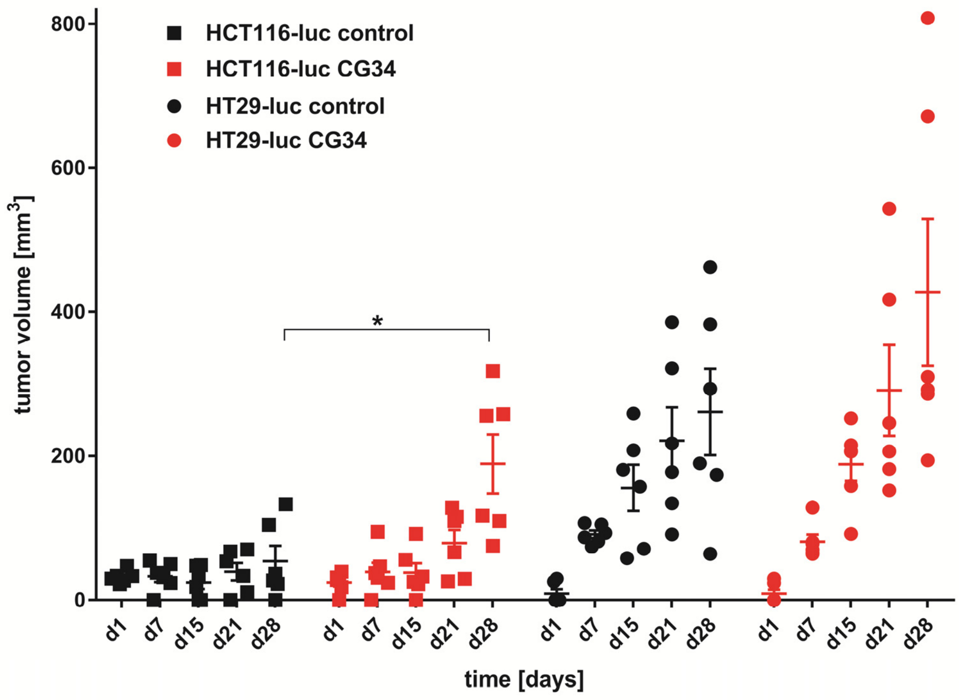

Figure S2. Tumor volume data of the treatment experiment shown in Figure 5B, but with individual data points and grouped by tumor and treatment type.

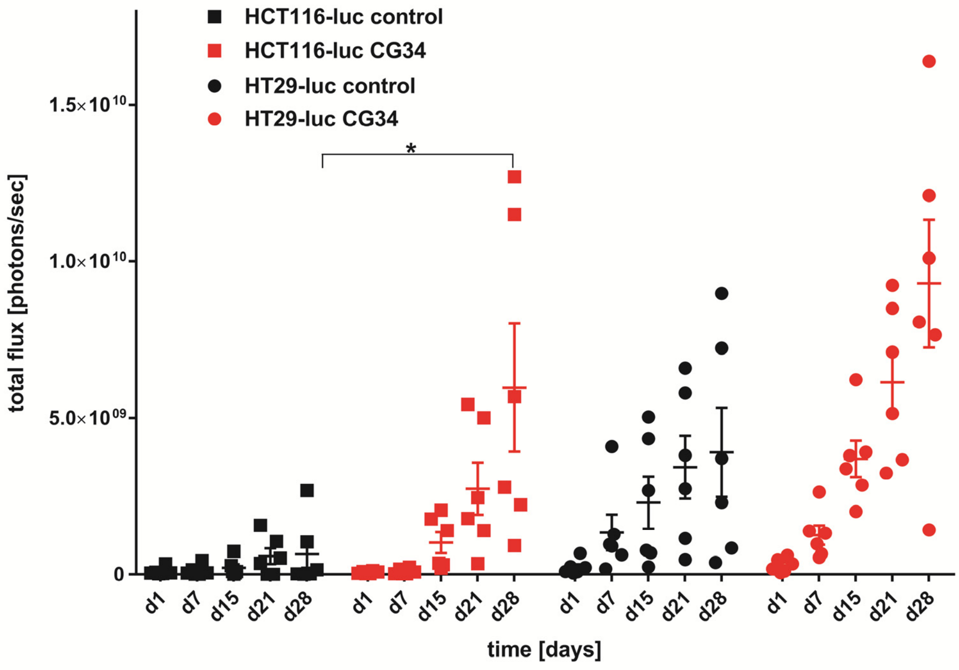

Figure S3. Bioluminescence data of the treatment experiment shown in Figure 5B, but with individual data points and grouped by tumor and treatment type.
